# New ST623 of *Cryptococcus neoformans* isolated from a patient with non-Hodgkin’s lymphoma in the Brazilian Amazon

**DOI:** 10.1186/s12941-020-00361-3

**Published:** 2020-05-20

**Authors:** Lucyane Mendes Silva, William Antunes Ferreira, Roberto Alexandre Alves Barbosa Filho, Marcus Vinícius Guimarães Lacerda, Guilherme Motta Antunes Ferreira, Maria de Nazaré Saunier, Marielle Machado Macedo, Denise de Almeida Cristo, Marla Jalene Alves, Ani Beatriz Jackisch-Matsuura, Cristina Motta Ferreira

**Affiliations:** 1 Fundação Hospitalar de Hematologia e Hemoterapia do Amazonas–HEMOAM, Manaus, AM Brazil; 2 Universidade do Estado do Amazonas-UEA, Manaus, AM Brazil; 3 Fundação de Dermatologia Tropical e Venereologia Alfredo da Matta-FUAM, Manaus, Brazil; 4 Universidade Federal do Amazonas-UFAM, Manaus, AM Brazil; 5 Fundação de Medicina Tropical Dr. Heitor Vieira Dourado, Manaus, AM Brazil; 6 Instituto Leônidas e Maria-Deane, FIOCRUZ, Manaus, AM Brazil

**Keywords:** Phylogeny, *C. neorformans*, MLST, Non-Hodgkin disease, ST623

## Abstract

**Background:**

Cryptococcosis is a disease of wide geographic distribution. It is most critical when it affects immunocompromised patients, with AIDS, tuberculosis or other diseases that require prolonged hospitalization.

**Methods:**

This study described a case report, molecular epidemiology, the phylogenetic relationship, along with antifungal susceptibility test of a new ST 623 of *C. neoformans* isolated in a patient with non-Hodgkin’s Lymphoma, from Manaus, Brazil.

**Results:**

The new *C. neoformans* was susceptible to all antifungal drugs tested. Our results showed that ST623 new clone has no evident evolutionary proximity to any other ST of the VNI subtype group identified in Brazil.

**Conclusions:**

In the context of phylogenetic analysis, this new genotype belongs to VNI subtype, and subsequencing complete genome studies are necessary to better understand the phylogenetic relationships amongst STs in this group.

## Background

Cryptococcosis is a serious disease possessing a wide geographic distribution, with a global burden of 957,900 cases of cryptococcal meningitis per year, resulting in 624,700 deaths [1]. This disease is an opportunistic mycosis caused by a complex called *Cryptococcus neoformans* and *C. gattii* [2], classified into four subtypes: VNI-VNII, VNIII, VNIV and VGI, VGII, VGIII, VGIV [3]. They are frequently isolated from bird excreta and trees hollows, penetrating the human host by inhaling infectious propagules [4], or through injured skin, causing different infections such as pulmonary cryptococcosis, nodules on the skin, meningitis or cryptococcal fungemia [3, 5–8].

In Latin America, there are records of 2400 deaths per year [9]. In countries like Colombia, the annual incidence is 2.4/cases/10^6^ inhabitants. In Mexico, the prevalence in patients with immunosuppression caused by different diseases is 21%; while in Venezuela it is 19%; and in Argentina, the prevalence is 20% [10]. In the city of Rio de Janeiro, Brazil, the annual incidence of cryptococcal meningitis is 0.45 cases/10^6^ inhabitants, while in Northeastern Brazil; it is considered endemic [9]. Data from the Brazilian Ministry of Health evidence annual incidence of 7000 cases of cryptococcal meningoencephalitis [11].

As it is considered an opportunistic mycosis, and potentially severe in certain patients, the availability of more sensitive tests for an enhanced routine laboratory diagnosis and treatment efficacy are indispensable for the proper control and monitoring of cryptococcosis. This rapid communications describes the molecular epidemiology, the phylogenetic relationships and the results of antifungal susceptibility test of a new ST (Sequencing Typing) from *C. neoformans* (ST 623).

This study was approved by the Foundation Human Research Ethical Committee (CEP/HEMOAM) under CAAE Nº 73548017.5.0000.0009, and all methods were performed in accordance with the relevant guidelines and regulations. Patients and a parent or guardian of any child participant enrolled in the study provided their written informed consent, before specimens were collected and the results and data were used for the management of each respective patient.

## Case report

A 72 years old, retired farmer, living in the city of Manaus-Amazon-Brazil, sought care at the State Center of Reference in Dermatology—”Alfredo da Matta” Foundation; reported that he observed a tumor approximately 4 cm in diameter in the occipital region, among others distributed diffusely throughout the body. A posterior cervical lymph node biopsy was performed and he was diagnosed with non-Hodgkin lymphoma. During the same period, an abdomen scan was ordered, and he was referred to the Foundation Hospital of Hematology and Hemotherapy Blood Center of Amazonas (HEMOAM), a reference center for Onco-hematologic Diseases.

On May 06, 2017, he was treated at HEMOAM with generalized pruritus symptom and palpable liver. He was medicated with antihistamine and antipruritic. On July 06, 2017 after consultations with hematologists, blood tests were performed complementary to the diagnosis to start chemotherapy of LYMPHOMA. On Dezember 06, 2017 the patient presented: abdominal computed tomography (TC Abd): cervical region with expansive/infiltrative lymph node mass, determining almost complete thrombosis of the left internal jugular vein + pulmonary emphysema + multiple mediastinal and axillary lymph node enlargement involving visceral/level and left internal mammary chain, retroperitoneal and mesenteric, inguinal, thoraco-lumbar and internal jugular vein thrombosis (tumor compression); and Hepatitis C (HCV) reagent. The patient was submitted to chemotherapy protocol from August 08, 2017 until October 10, 2017.

Still under the chemotherapy protocol for lymphoma, the patient presented a fever 37.5 °C (99.5 °F), coryza, sneezing, and elevation of transaminases with rates three times above normal limits. Subsequently, he attended the HEMOAM emergency room reporting weakness, a febrile peak of 38 °C (100.4 °F), vertigo, lack of appetite, nausea and epigastralgia. The laboratory findings were as follows: Haemoglobin (Hb): 12.6 g/L; Hematocrit (Ht) 36.8%; Platelet: 101,000/mm^3^; neutrophil: 9.8%; leucocyte: 2400/mm^3^; positive blood culture *for Cryptococcus neoformans*, and the diagnosis of Aplasia after chemotherapy. The patient was admitted for parenteral treatment with the antibiotics cefepime + clarithromycin and blood cultures were performed. However, due to his worsening clinical conditions, he was transferred to a University Hospital in Manaus on November 06, 2017.

Remaining in the ward of the University Hospital, the patient started treatment with Fluconazol 200 mg for 14 days, after culture result identifying the presence of yeasts. The patient continued in the ward, with worsening clinical evolution of: pulmonary infection + Neurological syndrome (seizure absence? + disorientation + cognitive alteration) + Hydroelectrolytic disturbance (hypokalemia) + Intestinal constipation + Plaquetopenia. Cefepime and Clarithromycin empirically therapy was discontinued, substituted by the Meropenem antibiotic. From November 07, 2017 to November 09, 2017 the clinical condition evolved to cardio-respiratory arrest in Asystole, followed by resuscitation and transferred to the Intensive Care Unit (ICU); still critical under antibiotic therapy, another cardiac arrest on November 09, 2017 and, due to respiratory septic shock, evolved to death.

## Materials and methods

### Laboratorial identification and antifungal susceptibility test

Following the two positives blood cultures (BACT/ALERT FA PLUS, Biomérieux, Brasil), the subculture was carried out in modified Sabouraud dextrose agar medium [12] and later in the media of canothothin-glycine blue bromothymol (CGB) and Niger Seed Agar for species differentiation. The phenotypic identification and minimum inhibitory concentration (MIC) values for fluconazole, amphotericin B and flucytosine were performed using VITEK-2 Compact equipment (bioMerieux, Brazil). Aliquots of the *C. neoformans* were stored at − 80 degrees Celsisus (°C), in a cryotube with Brain Heat Infusion Broth (BHI) (Himedia, Hexasystens-Mumbai, India) + 20% Glycerol for further molecular testing.

### Determination of molecular type

DNA was extracted using DNeasy Blood & Tissue Kit (Qiagen, Hilden, Germany) according to the manufacturer´s instructions. The molecular identification of the fungus was determined applying the enzymatic restriction protocol (PCR–RFLP). The reaction for amplification of the gene was carried out in a reation volume of 25 µl. Each reaction contained 2 µl of genomic DNA extract (28 ng); 2.5 µl of 10× PCR buffer; 2.5 µl of MgCl2 (50 mM); 5 µl of DNTp mix (2.5 µM) (Invitrogen, Carlsbad, CA, USA); 0.5 µl of the primers FW-5´-ATGTCCTCCCAAGC CCTCGACTCCG-3´; *SJ*01-5´-TTAAGACCTCTGAACACCGCTCC-3´ (10 µM) and 0.5 µl of Taq DNA polymerase (5 U/ml) (Invitrogen), Martins et al. [4]. Reactions were performed on a thermal cycler (Proflex PCR system, Applied Biosystems, Foster City, CA) at the following conditions: Initial denaturation at 95 °C for 5 min, followed by 40 cycles at 95 °C for 5 min; 95 °C for 45 s; annealing at 55 °C for 45 s (ranging from 55 °C, 56 °C, 56.5 °C to 57 °C), extension at 72 °C for 2 min, then final extension at 72 °C for 7 min, Martins et al. [4].

The PCR product was demonstrated by SYBR™ safe (Invitrogen) DNA gel electrophoresis on 1.5% agar gels. Following this, sequencing was performed using the ABI PRISM 3130 XL Genetic Analyzer (Applied Biosystems, Foster City, CA), according to manufacturer’s instructions. Sequences generated in the forward and reverse directions were read using Geneious v.11 software. A consensus sequence was extracted after being checked for discrepancies or mutations. The results were compared with the genomic DNA deposited in the site database (http://mlst.mycologylab.org/). The analysis of Multilocus Sequence Typing (MLST) for *C. neoformans* was performed according to the site protocol (http://mlst.mycologylab.org/).

Sequences of the MLST genes from *C. neoformans*, isolated from HEMOAM, were compared with other VNI subtypes sequences, selected due to genetic proximity criterion that ST623 has with this group. The sequences of the subtype are deposited in the MLST database (http://mlst.mycologylab.org), totaling 174 samples. The nucleotide sequences of the seven alleles of the 174 samples were edited and aligned by the MEGA X program using the MUSCLE tool [13, 14]. The alignenment were analysed using MEGA X and DnaSP 6.0 programs. The phylogenetic tree were edited using ITOL program (https://itol.embl.de) [15]. To reconstruct the phylogenetic relationship between STs and VNI subtypes, the sequence of the seven MLST markers were concatened and analysed to choose the evolutive model ‘Kimura 2 parameters’ for analysis, with gamma distribution and invariable substitution rates. One tree underwent 1000 resamples per bootstrap. Pearson’s correlation coefficient (r) was used to analyze the correlation between nucleotide diversity and the number of MLST alleles, as well as the number of haplotypes and the MLST alleles.

## Results and discussion

The microbiological test realized identified *Cryptococcus neoformans*. MIC showed susceptibility to all antifungal tested, with values of 1 µg/ml for fluconazole, 2 µg/ml for flucytosine and 0.5 µg/ml for amphotericin B. PCR–RFLP protocol identified the molecular type VNI, and comparative analyzes with the sequences deposited on the MLST website, made it possible to identify a new clone of *Cryptococcus neoformans***ST623**. GenBank accession numbers of the *C. neoformans* allele from our case are MN065812, MN065813, MN065814, MN065815, MN065816, MN065817, and MN065818.

**Fig. 1:**
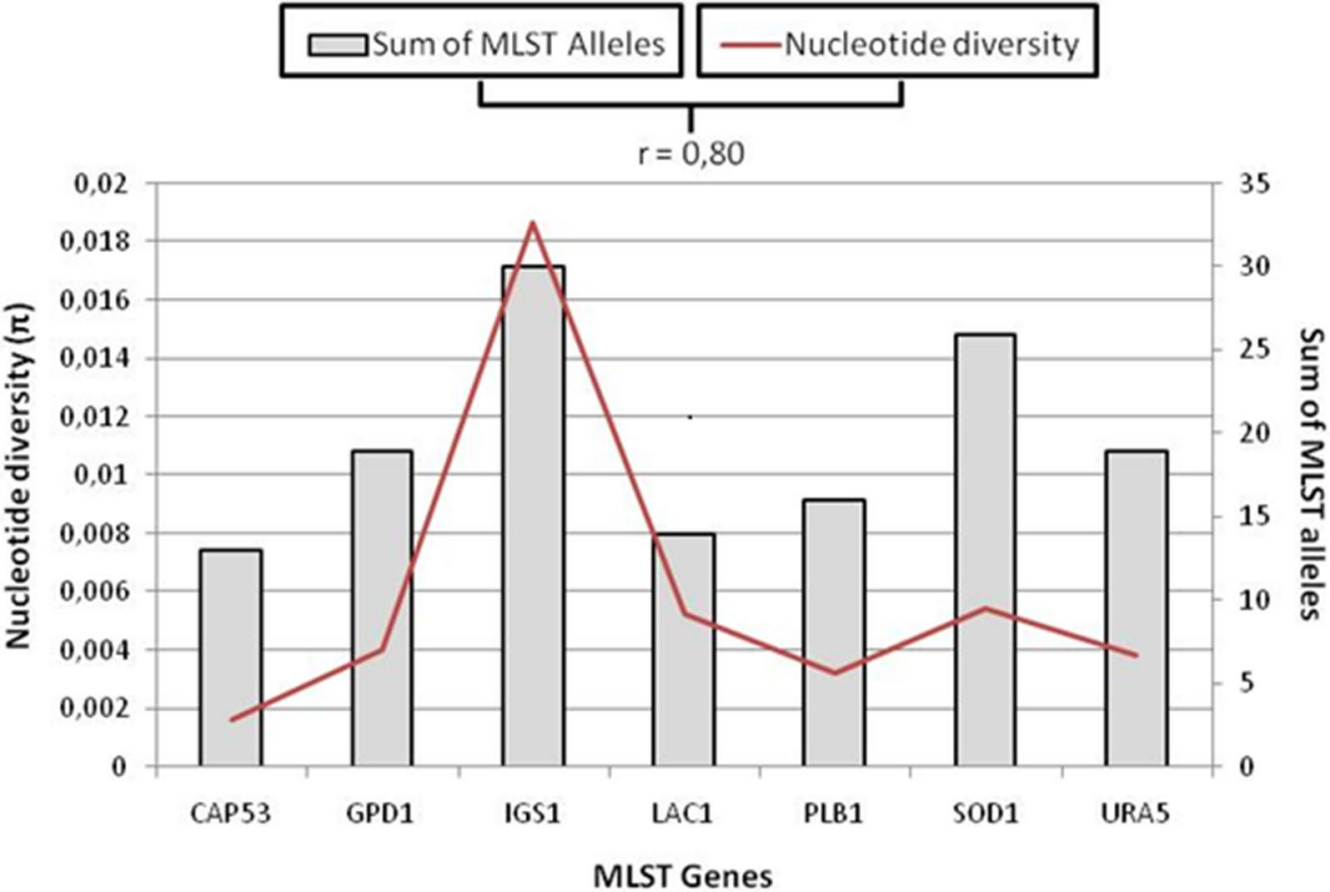
Allelic diversity of VNI group

It was also observed that nucleotide diversity (Fig. 1) was higher when compared to all *C. neoformans* STs results studied by Muñoz et al. [16]. Of the seven genes analyzed, IGS1 presented the highest number of alleles (30) and highest nucleotide diversity (0.01864). The CAP53 presented the lowest values of alleles (13) and nuclotide diversity (0.0162).

A strong positive correlation of r = 0.80 was observed between nucleotide diversity and the number of MLST alleles, as well as the number of haplotypes and the MLST alleles (r = 0.85). The fact that IGS1 corresponds to an intergenic region, contributes to the high nucleotide diversity observed in this region, characterizing it as a hypervariable marker, as described by Muñoz et al. [16, 17] (Fig. 1).

**Fig. 2:**
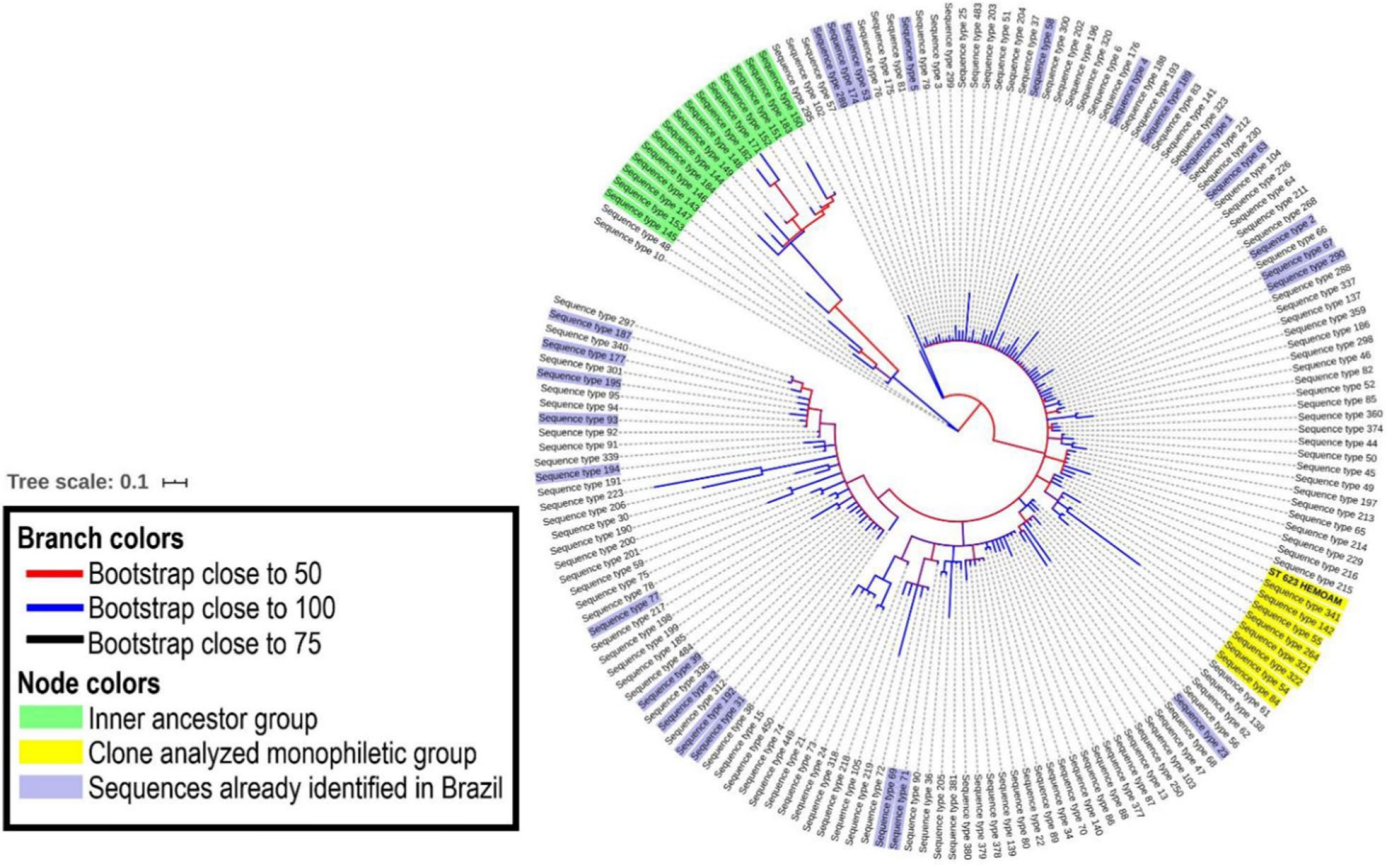
Phylogenetic tree of the VNI subtype STs Groups. Branches in blue color represents groups with bootstrap near to 100. Branches in red color represents groups with bootstrap near to 50. The monophyletic group, containing **ST623**, is in yellow, and the green one is the internal ancestor group of the VNI subtype

The analysis performed with the selected sequences for the evolutionary study of the new ST623 showed the ancestral relationship between monophyletic group (yellow) and the others groups studied. It was observed the proximity of ST623 and STs 341,142, 55, 264, 322, 54 and 84, where these topologies are statistically supported by the high bootstrap values. It was also possible to reconstruct the ancestral relationship of the others monophyletic groups (green) formed by STs 145,153,147,143,145,184,144,149,148,182,171,152,151,183,150 and the others STs of the VNI subtype. Our results showed also that ST623 clone, has no evident evolutionary proximity to any other ST of the VNI subtype group, identified in Brazil by Rocha et al. and Ferreira-Paim et al. [11, 18], from clinical and environmental samples, as can be observed in Fig. 2.

## Conclusions

The new ST623 genotype was isolated from a patient with non-hodgkin´s lymphoma, which evolved to death. In the evolutionary context of phylogenetic analysis, this genotype belongs to VNI subtype; however, the relationships between the STs in this group only can be elucidated after an approach with complete genome sequencing (WGS) studies.

## Data Availability

All data generated or analysed during this study are included in this published article Not applicable.
